# Is there an association between lateral uterine localization of the placenta and pregnancy outcomes?

**DOI:** 10.1007/s00404-024-07910-7

**Published:** 2025-01-21

**Authors:** Hanoch Schreiber, Gal Cohen, Ofer Markovitch, Omer Weitzner, Sivan Farladansky-Gershnabel, Tal Biron-Shental, Michal Kovo

**Affiliations:** 1 Department of Obstetrics and Gynecology, Meir Medical Center, 59 Tchernichovsky St., 44281 Kfar Saba, Israel; 2 School of Medicine, Faculty of Medical and Health Sciences, Tel Aviv University, Tel Aviv, Israel; 3 Department of Research, Meir Medical Center, Kfar Saba, Israel

**Keywords:** Placenta location, Lateral placentation, Retained placenta, Adverse perinatal outcomes

## Abstract

**Purpose:**

To evaluate the association between lateral placentation and adverse perinatal outcomes, including rates of small for gestational age (SGA) neonates, hypertensive (HTN) disorders, and preterm delivery, as well as postpartum hemorrhage and retained placenta.

**Methods:**

This retrospective cohort study included all women with singleton pregnancies who underwent a trial of labor after reaching 24 weeks of gestation, at a single tertiary medical center, over a period of 6 years. The study group included women with lateral placentation. Controls were women with anterior, posterior, or fundal placentation. Power analysis indicated that 882 women in each group would be sufficient to detect an increased rate of the primary outcomes: preterm delivery, hypertensive disorders or SGA in the lateral placenta group. Secondary outcomes were Apgar score, cord pH and retained placenta.

**Results:**

Overall, 1,817 (7.6%) women had lateral placenta and 21,991 (92.4%) anterior, posterior, or fundal placentation. No significant differences were observed between groups in the rates of hypertensive disorders, SGA or preterm birth. Lateral placentation was associated with a longer third stage of labor (11.1 ± 8.6 min vs. 10.4 ± 7.2 min, p = 0.001) and higher rate of retained placenta (5.7% vs. 4.2%, p = 0.002). Multivariate regression found that lateral placentation was independently associated with longer third stage of labor.

**Conclusion:**

Lateral placentation was not associated with increased rates of hypertensive disorders, preterm birth or SGA infants. It was linked to a longer third stage of labor but without a significant impact on maternal or perinatal complications.

## What does this study add to the clinical work

**Table:** 

Lateral uterine location of the placenta is not associated with adverse maternal or neonatal outcomes.	

## Introduction

Appropriate placental development is critical for a healthy pregnancy, facilitated by various autocrine, endocrine and paracrine mechanisms that significantly impact fetal growth and maternal outcomes [1–4]. Proper functioning of the placenta is essential for normal pregnancy progression, and disruptions can lead to adverse fetal and maternal outcomes [5–8].

During human placentation, the blastocyst typically implants in the upper portion of the uterus, with the placenta most often developing on the anterior or posterior uterine wall [9, 10]. However, implantation on the lateral uterine walls, which differ in anatomical curvature and blood supply, may influence pregnancy outcomes. Conditions like placenta previa and low lying placenta that are associated with atypical placental positioning, are well-known risk-factors for adverse outcomes such as maternal hemorrhage, fetal growth restriction and increased placental vascular mal-perfusion lesions [11–13].

Lateral placentation occurs in approximately 12% of pregnancies [14]. While the effects of lateral placental location on pregnancy outcomes have been studied, results remain controversial. Some studies found that placental location did not have a significant effect on pregnancy outcomes [15–17]. However, a meta-analysis [18] of 15 cohort studies reported an association between lateral placental location and increased risks of preeclampsia, hypertensive disorders, preterm birth and small for gestational age (SGA) infants, suggesting that lateral placentation is linked to increased risk of placenta-related pregnancy complications.

Despite these findings, consensus on the impact of lateral placentation is lacking, largely due to the small size and single-center nature of most studies. Therefore, this study evaluated the association between lateral placental location and delivery outcomes, particularly those potentially related to impaired placental function.

## Methods

This retrospective cohort study included all women with singleton pregnancies who delivered vaginally after 24 weeks of gestational at a single tertiary care medical center from January 2014 to October 2020. Women with lateral placental implantation (lateral placenta group) were compared to those with anterior, posterior, or fundal implantations (control group). Placental location was determined for all patients via transabdominal ultrasound upon admission to the delivery ward.

Exclusion criteria were elective cesarean delivery (CD), multiple gestations and pregnancies with low lying placenta or placenta previa. Cases of intrauterine fetal demise, known structural uterine anomalies, fetal chromosomal aberrations and those with missing data regarding placental location were excluded from the analysis.

### Data collection

Data were retrieved from the electronic medical records of the parturients and neonates. Maternal baseline parameters and medical history including maternal age, gestational age at delivery, gravidity, parity, smoking status, body mass index (BMI, kg/m^2^), and the presence of hypertensive disorders (chronic hypertension, gestational hypertension and preeclampsia), as well as pre-gestational diabetes mellitus (DM) and gestational DM, were collected.

Birth and delivery outcomes included spontaneous vs. induced labor, use of epidural anesthesia, presence of meconium, duration of the second and third stages of labor, retained placenta and estimated maternal blood loss during labor. Retained placenta was defined as a placenta that failed to separate within 30 min after fetal extraction, or when manual extraction of the placenta was required due to retained placenta.

Neonatal outcomes were also retrieved from the electronic records, including neonatal weight, Apgar scores, cord pH, neonatal intensive care unit (NICU) admissions, neonatal hypoglycemia, respiratory distress with mechanical ventilation and SGA defined as birthweight < 10th percentile for gestational age according to the local growth charts [19]. Placenta location was analyzed by ultrasound at presentation for delivery.

Baseline characteristics, pregnancy, labor and delivery outcomes were compared between the groups.

### Statistical analysis

Statistical analyses were performed with SPSS-25 software (IBM Corp., Armonk, NY, USA). Nominal data were described as numbers and percentages. Continuous variables were described by means and standard deviations. Metric variables were analyzed with t-test or Mann–Whitney test. Discrete variables were analyzed using chi squared. A P-value < 0.05 was considered statistically significant. Multivariable logistic regression and adjusted odds ratios (OR) were calculated to identify risk-factors associated with lateral placentation and adverse outcomes.

### Power analysis

We found that approximately 3.5% of the women in the study were diagnosed with hypertension or preeclampsia. To test the hypothesis that there is difference between study groups manifested by an increase in the rate of hypertensive disorders, we made a clinical assumption that a two-fold increase in the rate of hypertensive disorders from 3.5% to 7%, would be considered a significant clinical difference. Based on this clinical assumption, the power analysis calculated that at least 636 women in each group would be sufficient to detect a significant increase in the rate of pre-term delivery, hypertensive disorders or SGA in the lateral placenta group, under the assumptions of a type I error of 5% and at least 80% power.

## Results

**Fig. 1:**
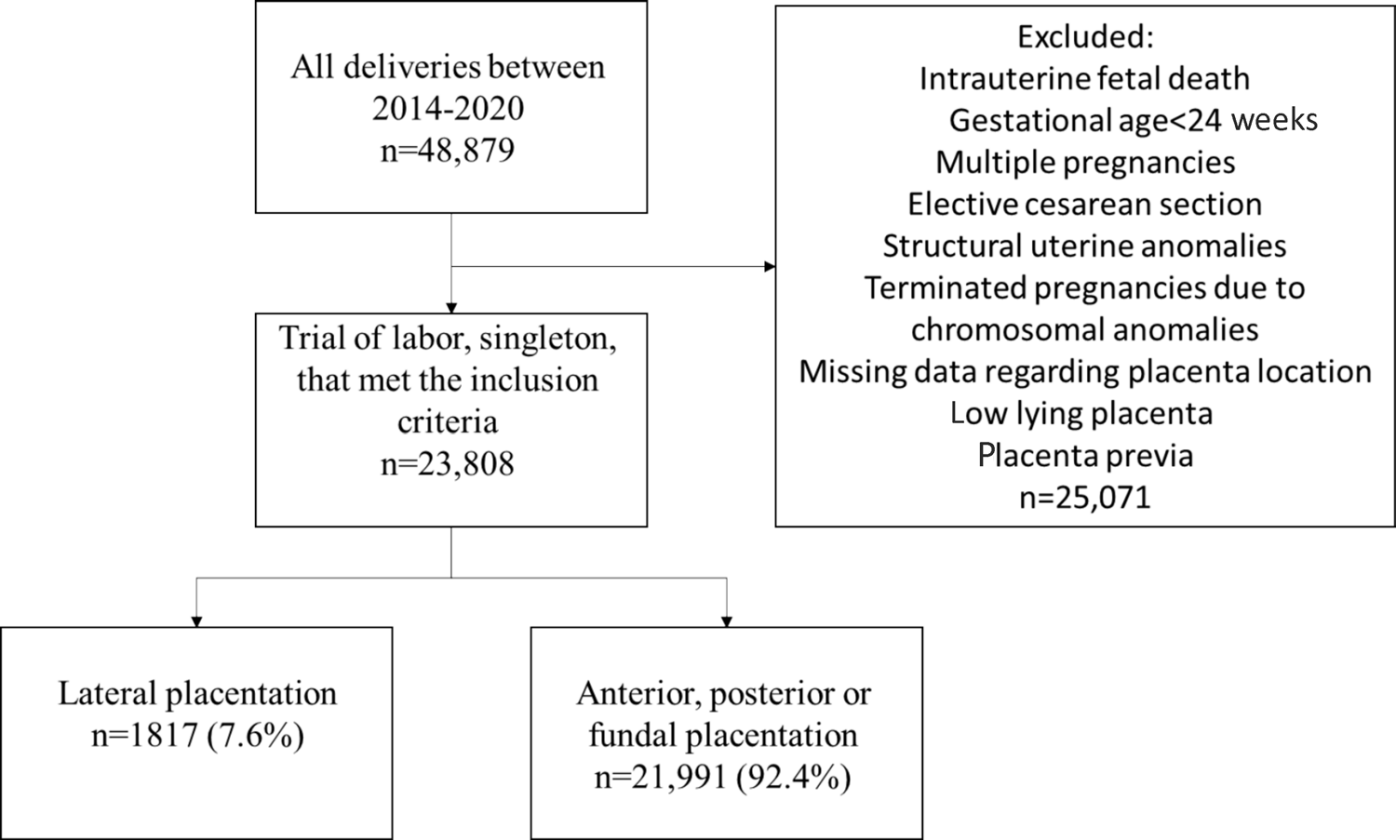
Study flow diagram

During the study period, there were 48,879 deliveries at our institution, of which 25,071 met the exclusion criteria. Of the remaining 23,808 women, the placenta was located laterally in 1817 (7.6%) and 21,991 (92.4%) constituted the control group with anterior, posterior, or fundal placental location (Fig. 1).

Table 1 presents maternal characteristics of the study groups. As compared to controls, the lateral placenta group was older, 31.1 ± 5.5 years vs. 30.8 ± 5.3 years (p = 0.021) and fewer was nulliparous, 39.1% vs. 43.3%, respectively, p = 0.001. There were no between group differences in gestational age at delivery, maternal BMI, rate of vaginal birth after cesarean, smoking, DM, hypertensive disorders or oligohydramnios.

**Table 1:** Baseline characteristics in relation to lateral placentation

Variable*	Lateral placentation (n = 1817)	Control (n = 21,991)	P-value
Maternal age, years	31.1 ± 5.5	30.8 ± 5.3	0.021
Gestational age (weeks + days)	39 + 3 ± 2	39 + 2 ± 2	0.107
Preterm birth before 37 weeks	73 (4.0)	955 (4.3)	0.513
Nulliparity	711 (39.1)	9525 (43.3)	0.001
Body mass index, kg/m^2^	24 ± 5	24 ± 5.1	0.810
DM	180 (9.9)	2481 (11.3)	0.074
Smoking (n, %)	95 (5.2)	1198 (5.4)	0.692
Vaginal birth after cesarean delivery (n, %)	104 (5.7)	1465 (6.7)	0.640
Hypertensive disorders	65 (3.6)	793 (3.6)	0.942
Oligohydramnios	51 (2.8)	584 (2.6)	0.704
Male fetus	892 (49.1)	11,116 (50.5)	0.234

*Data are presented as n (%) or mean ± SD. DM includes pre-gestational and gestational diabetes, Hypertensive disorders include gestational and chronic hypertension and preeclampsia

Labor and delivery characteristics are presented in Table 2. The lateral placenta group was characterized by lower rate of CD (p = 0.003), longer third stage of labor (p = 0.001) and increased rate of retained placenta (p = 0.002), as compared to the control group. The rates of induction of labor, meconium, epidural anesthesia, operative vaginal delivery, nuchal cord, true knot were similar between the groups.

**Table 2:** Labor and delivery characteristics in relation to lateral placentation

Characteristic*	Lateral placentation (n = 1817)	Control (n = 21,991)	P-value
Induction of labor	460 (26.2)	5761 (27.2)	0.358
Meconium	215 (13.9)	2700 (14.4)	0.725
Epidural	1326 (73)	16,373 (74.4)	0.168
Vacuum delivery	165 (9.1)	2251 (10.2)	0.117
Cesarean delivery	134 (7.4)	2086 (9.5)	0.003
Second stage duration, min	60 ± 68	66 ± 70	0.001
Third stage duration, min	11.1 ± 8.6	10.4 ± 7.2	0.001
Retained placenta	104 (5.7)	926 (4.2)	0.002
Nuchal cord	481 (28.3)	2635 (27.8)	0.628
True knot	27 (1.6)	300 (1.5)	0.716

*Data are presented as n (%) or mean ± SD

Table 3 presents neonatal outcomes of the study groups. There were no between group differences in the rate of low Apgar scores, cord pH < 7.1, NICU admission, neonatal hypoglycemia, need for mechanical ventilation or SGA.

**Table 3:** Neonatal outcomes of the study groups

Outcome*	Lateral placentation (n = 1817)	Control (n = 21,991)	P-value
Birth weight, g	3244 ± 461	3255 ± 479	0.362
5-min Apgar ≤ 5	6 (0.33)	47 (0.21)	0.311
pH ≤ 7.1	27 (1.48)	301 (1.36)	0.265
Estimated maternal blood loss, ml	296 ± 194	290 ± 188	0.218
Maternal blood transfusion	15 (0.8)	153 (0.64)	0.525
NICU	45 (2.47)	464 (2.11)	0.693
Neonatal hypoglycemia	6 (0.33)	128 (0.58)	0.156
Respiratory distress with mechanical ventilation	7 (0.38)	77 (0.35)	0.844
Small for gestational age	157 (8.6)	1855 (8.4)	0.761

*Data are presented as n (%) or mean ± SD

### Multivariable logistic regression

Multivariate logistic regression was performed to assess the association between lateral placentation and various outcomes, adjusting for factors found to be significant in univariate analysis, including maternal age, nulliparity, cesarean delivery (CD), retained placenta and duration of the second and third stages of labor. Lateral placentation was independently associated with lower rate of CD, and shorter duration of the second stage of labor. Lateral placentation was found to be an independent risk factor for a prolonged third sate of labor (Table 4).

**Table 4:** Multivariate analysis model for factors associated with lateral placenta

Variable	P-value	Odds ratio	95% confidence interval	
			Lower	Upper
Maternal age	0.244	1.006	0.996	1.018
Nulliparity	0.571	0.959	0.831	1.108
Cesarean delivery	**0.013**	2.368	1.201	4.67
Retained placenta	0.129	1.225	0.943	1.591
Second stage duration	**0.037**	0.937	0.882	0.996
Third stage duration	**0.02**	0.940	0.884	0.998

## Discussion

The present study did not find significant associations between lateral placental location and placental-related pregnancy complications, such as hypertensive disorders, preterm birth, or SGA neonates. The hypothesis linking lateral placentation to adverse pregnancy outcomes assumes that the lateral uterine walls receive their primary blood supply from the ipsilateral uterine artery. Consequently, this could lead to reduced uterine blood flow and impaired placental perfusion compared to placentas situated on the anterior or posterior uterine walls [18, 20]. Impaired placental perfusion has been strongly associated with abnormal placentation and complications such as maternal hypertensive disorders, fetal growth restriction, and preterm birth [13, 16].

However, the lack of association between lateral placentation and these complications, as demonstrated in the present study, suggests that placental perfusion is not notably compromised in these cases. This is supported by inconsistent reports in the literature regarding lateral placentation and its potential link to pregnancy complications. Notably, studies vary in their definitions of SGA and in their comparison groups, which may explain the heterogeneity of the findings. For example, Granfors et al. compared lateral placentation specifically to posterior placentation, defining SGA as birthweight more than two standard deviations below the mean for gestational age and sex, reporting a 4.9% SGA rate in the lateral group compared to 2.9% in the posterior placenta group [21]. In contrast, another study excluded fundal placentation from the "central" group (anterior and posterior placentas), defining SGA as birthweight below the 10th percentile, and reported a SGA rate of 19.6% in the lateral placentation group vs. 11.6% in the “central” group [16]. The present study, in contrast, classified placental location based on a biological principle related to uterine blood supply patterns, comparing lateral placentas to a combined group of anterior, posterior, and fundal placentas. This approach addresses the blood supply differences more comprehensively by focusing on the fact that lateral placentation relies primarily on one uterine artery, while central placentation benefits from a more distributed blood supply.

Consistent with previous studies, we observed certain maternal and obstetric characteristics more frequently in women with lateral placentation, including advanced maternal age [16, 21, 22] and lower rates of nulliparity [17]. Women with lateral placentas also had slightly longer second and third stages of labor. Although these differences were statistically significant, the clinical relevance of a few minutes’ difference in labor duration is minimal. Importantly, there was no increase in maternal blood loss or need for postpartum blood transfusion in cases with lateral placentation.

The strengths of this study include the large sample size, uniform treatment protocols, and comprehensive documentation, which allowed us to control for multiple confounders and conduct a more accurate assessment of the potential impact of lateral placental localization. By basing our analysis on the distinct patterns of uterine blood supply, particularly the reliance on a single uterine artery for lateral placentation, we provided a biologically sound framework for comparison. Nevertheless, this study had limitations, primarily due to its retrospective design and the reliance on coding for diagnosis, which may introduce misclassification bias. Information on previous uterine surgery and endometriosis were not in the database, However, as elective cesarean sections were not part of this study, cases where the decision to perform a planned cesarean was influenced by this information were not included in the analysis. Although the database did not contain information on maternal anemia, in most instances where iron deficiency anemia required intervention, it was provided prior to delivery.

In conclusion, lateral placentation does not appear to increase the risk of placenta-related pregnancy complications such as hypertensive disorders, SGA, or preterm birth, compared to central placentation. Further prospective studies are warranted to validate these findings and to explore other potential factors influencing placental perfusion and pregnancy outcomes in cases of lateral placentation.

## Data Availability

The data that support the findings of this study are available on request from the corresponding author, upon reasonable request.
